# Only the Good Die Old? Ontogenetic Determinants of Locomotor Performance in Eastern Cottontail Rabbits (*Sylvilagus floridanus*)

**DOI:** 10.1093/iob/obab037

**Published:** 2022-01-06

**Authors:** Jesse W Young, Adam D Foster, Gabrielle A Russo, Gregory A Smith, Michael T Butcher

**Affiliations:** Department of Anatomy and Neurobiology, Northeast Ohio Medical University (NEOMED), Rootstown, OH 44272, USA; Department of Anatomy, School of Osteopathic Medicine, Campbell University, Buies Creek, NC 27506, USA; Department of Anthropology, Stony Brook University, Stony Brook, NY 11794-4364, USA; Department of Biological Sciences, Kent State University at Stark, North Canton, OH 44720, USA; Department of Biological Sciences, Youngstown State University, Youngstown, OH 44555, USA

## Abstract

For many animals, the juvenile stage of life can be particularly perilous. Once independent, immature animals must often complete the same basic survival functions as adults despite smaller body size and other growth-related limits on performance. Because, by definition, juveniles have yet to reproduce, we should expect strong selection for mechanisms to offset these ontogenetic limitations, allowing individuals to reach reproductive adulthood and maintain Darwinian fitness. We use an integrated ontogenetic dataset on morphology, locomotor performance, and longevity in wild cottontail rabbits (*Sylvilagus floridanus*, Allen 1848) to test the hypothesis that prey animals are under selective pressure to maximize juvenile performance. We predicted that (1) juveniles would accelerate more quickly than adults, allowing them to reach adult-like escape speeds, and (2) juveniles with greater levels of performance should survive for longer durations in the wild, thus increasing their reproductive potential. Using high-speed video and force platform measurements, we quantified burst acceleration, escape speed, and mechanical power production in 38 wild-caught *S. floridanus* (26 juveniles, 12 adults; all rabbits >1 kg in body mass were designated to be adults, based on published growth curves and evidence of epiphyseal fusion). A subsample of 22 rabbits (15 juveniles, 7 adults) was fitted with radio-telemetry collars for documenting survivorship in the wild. We found that acceleration and escape speed peaked in the late juvenile period in *S. floridanus*, at an age range that coincides with a period of pronounced demographic attrition in wild populations. Differences in mass-specific mechanical power production explained ∼75% of the variation in acceleration across the dataset, indicating that juvenile rabbits outpace adults by producing more power per unit body mass. We found a positive, though non-significant, association between peak escape speed and survivorship duration in the wild, suggesting a complex relationship between locomotor performance and fitness in growing *S. floridanus*.

## Introduction

Juvenility can be particularly precarious for many animals (Williams 1966). Juveniles occupying overlapping ecological niches with adults must compete for the same resources and evade the same predators. Nevertheless, smaller body size, reduced muscle mass, lack of complete sensory-motor integration, and general musculoskeletal immaturity may limit performance capacity, endangering juveniles through increased predation risk and intraspecific resource competition with larger, more competent conspecifics (Carrier 1996). As a result, mass-specific mortality rates are often greater during the juvenile period than during adulthood, exerting significant influence on the pace of growth and other aspects of life history (Case 1978; Promislow and Harvey 1990). We should therefore expect behaviorally precocial, fast-growing prey animals to be under selection for improved capacities associated with predator escape, such as sprint speed, acceleration capacity, and jump distance (Herrel and Gibb 2006). For example, compared to adult conspecifics, immature mammals often have greater limb muscle mechanical advantage for enhancing force production and relatively robust long bones for enhancing bending load resistance (Carrier 1996; Currey 2002; Foster et al., 2019). Selection for such performance enhancing mechanisms should be particularly strong in small mammals, where juvenile survivorship is typically low and where population growth rates closely track variation in juvenile survivorship (Gaillard and Yoccoz 2003). Though heightened levels of locomotor performance have previously been documented in a broad spectrum of juvenile animals (Herrel and Gibb 2006), no study to date has holistically examined associations among morphology, performance and fitness in growing prey animals to formally test the adaptive significance of the morphological and behavioral traits that are thought to promote juvenile survival via enhanced performance capacities (Arnold 1983).

**Fig. 1:**
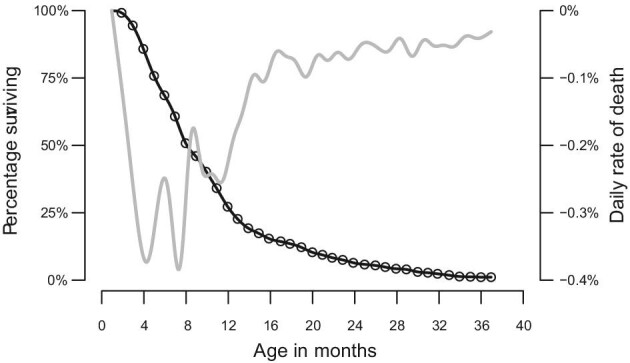
Survivorship of *S. floridanus* as a function of age; data from Lord Jr (1961). The demographic survivorship curve for is represented by the bold black line. Note the convex profile diagnostic of a type III survivorship curve, characterized by high mortality at young ages, and comparatively low mortality for the subset of individuals surviving this demographic bottleneck. The lighter grey line represents the first derivative of this curve with respect to age in days, corresponding to the percent of individuals in the cohort culled from the population at any given age (i.e., the “daily rate of attrition”).

Here, we use an integrated ontogenetic dataset on growth, locomotor performance, and longevity in eastern cottontail rabbits (*Sylvilagus floridanus*, Allen 1890) as a model system to test the hypothesis that prey animals are under selective predation pressure to maximize locomotor performance during the juvenile period. *Sylvilagus floridanus* is an ideal model in which to explore the degree to which natural selection might influence growth and locomotor performance across ontogeny: infants begin locomoting at 14–16 days, become independent juveniles at three weeks of age, and experience high predation pressure during the first year of life (Haugen 1942; Chapman et al. 1980). Eastern cottontail rabbits have many predators, including domesticated carnivores, wild carnivores, and raptors (Haugen 1942), and approximately 75% of individuals die by 12 months of age (Fig. 1), with more than 70% of annual mortality due to predation (Keith and Bloomer 1993*;*Fritsky 2006; Boland and Litvaitis 2008). Predator evasion is chiefly accomplished via quick evasive sprints to sites of refuge (i.e., undergrowth) (Trent and Rongstad 1974; Cowan and Bell 1986; Temple 1987). Diurnally active cottontails select microhabitats characterized by dense vegetation in which to hide, typically positioning themselves no more than 20 m from potential sites of refuge, and quickly accelerating to escape into refugia if a potential predator has been detected (Vidus-Rosin et al. 2010).

Our research focused on two specific aims. First, we investigated the ontogeny of locomotor performance in *S. floridanus*. We predicted that juvenile rabbits would accelerate more quickly than adults, allowing juveniles to achieve adult-like or greater escape speeds at the end of a stride, despite absolutely smaller body size. Following previous research on the biomechanics of acceleration (Roberts and Scales 2002; Williams et al. 2009), we also predicted that acceleration would be limited by mechanical power output, such that—for their body size—juvenile rabbits would be capable of greater power production than adults. Second, we tested for evidence of a relationship between locomotor performance (i.e., escape speed) and survivorship in juvenile *S. floridanus*, predicting that the juveniles that achieved the highest escape speeds would be better able to avoid predation and thus survive for longer durations in their natural habitat.

## Materials and Methods

All procedures were approved by the Northeast Ohio Medical University (NEOMED) Institutional Animal Care and Use Committee (Protocols 10-032 and 13-026, to JWY) and the Ohio Department of Natural Resources Division of Wildlife (Permits 14-310, 15-173, and 16-128 to GAS) prior to this research. Details of our data collection procedures have been published previously (Butcher et al. 2019; Foster et al. 2019) and will be only briefly summarized here.

### Animal sample

**Fig. 2:**
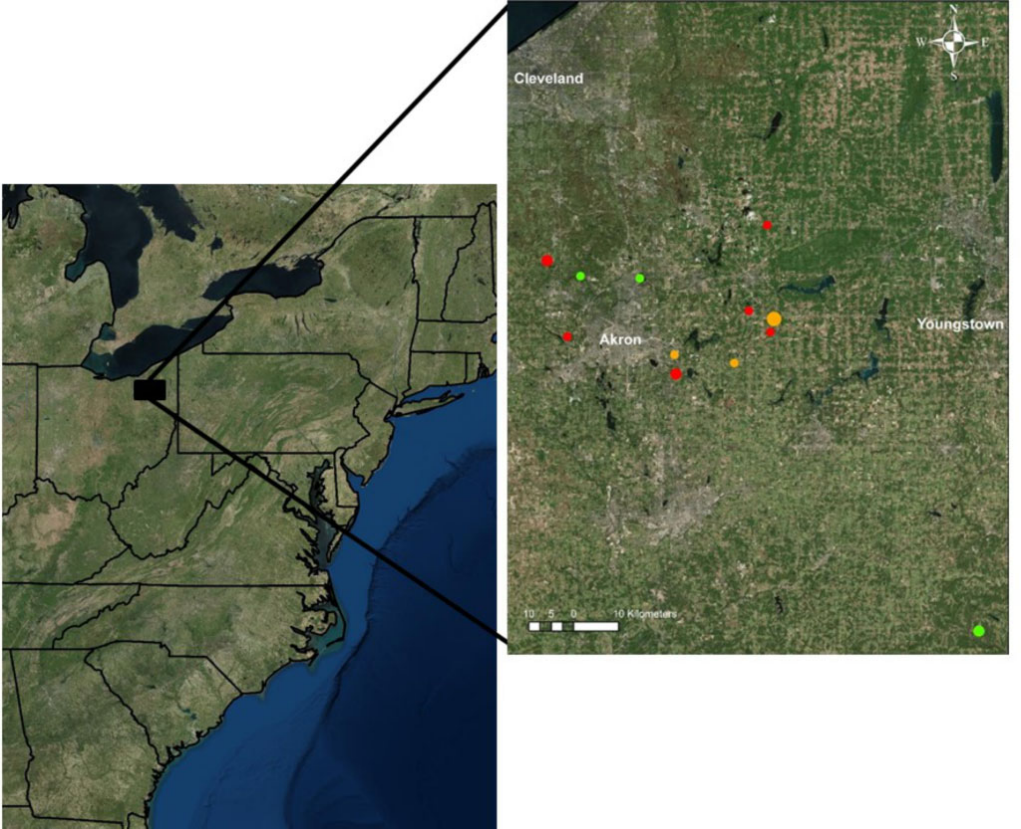
Map of locations in northeast Ohio where individual *S. floridanus* were trapped and either (1) released with attached radiocollars (red dots), (2) euthanized for muscle dissection (green dots), or (3) a combination of the two (orange dots). Marker diameter is proportional the number of rabbits trapped at each site. All trapping took place with prior permission and permits in Summit, Portage, and Columbiana counties (west to east on the map).

**Fig. 3:**
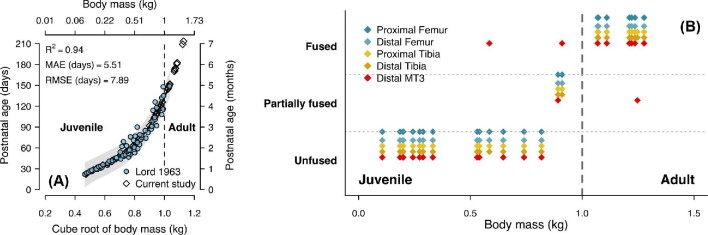
Validation of juvenile and adult age categories in the ontogenetic *S. floridanus* sample. (**A**) Relationship between age and body mass. Comparative model fitting established that age was best predicted by a cubic regression model of age in days versus the cube root of body mass in kg. Model fitting metrics are shown in the upper left corner of the plot (MAE: mean absolute error; RMSE: root mean squared error). The 95% prediction intervals (in gray) on the cubic regression model are drawn behind the points from the reference study (Lord Jr. 1963) and our current sample. Though the heaviest rabbits in our dataset extend beyond the body mass range of the original sample, the regression curve indicates that growth in mass has begun to plateau at these body sizes. The vertical dashed line drawn at 1 kg of body mass indicates the division between adult rabbits (>1 kg) and juvenile rabbits (<1 kg). (**B**) Age of epiphyseal fusion in the hindlimb skeleton of *S. floridanus.* Proximal and distal epiphyses in the femur, tibia, and third metatarsal were evaluated for fusion status in surface reconstructions of micro-commuted tomography (μCT) scans of limb bones collected from rabbits in the morphometric sample; see Young et al. (2014) for a description of μCT scanning methods. Growth plates were deemed unfused when we observed visible space between the diaphysis and epiphysis (indicating the present of radiolucent growth plate cartilage), partially fused when there was no visible space between the diaphysis and epiphysis, but an epiphyseal line was still visibly present, and fully closed when no epiphyseal line was evident. Note that the third metatarsal is characterized by a single epiphysis at the head (i.e., distal end). The vertical dashed line drawn at 1 kg of body mass indicates the division between adult rabbits (>1 kg) and juvenile rabbits (<1 kg).

Over three field seasons (2013–2015), we trapped a total of 61 *S. floridanus* rabbits at several sites in northeast Ohio where hunting was forbidden (Fig. 2). Due to variation in animal condition and temperament, we were able to collect locomotor performance data from 38 of these rabbits, 26 of whom were classified as juveniles and 12 as adults, based on body mass (M_b_) at the time of capture (i.e., individuals with a M_b_ <1 kg were designated “juveniles”; animals with a M_b_ >1kg were designated “adults”). The 1 kg body mass cutoff between age groups corresponds to a predicted age of 133 days (based on a published *S. floridanus* growth curve, Fig. 3A), which is roughly the age at which mass growth begins taper (Lord Jr. 1963) and long bone epiphyses fuse (Fig. 3B; see also Hale 1949). We did not separately identify males and females, given the difficulty in reliably sexing juvenile rabbits. However, *S. floridanus* shows limited sexual dimorphism (Olcott and Barry 2000). Of the 38 animals included in our locomotor performance sample, 22 (15 juveniles/7 adults) were fit with radiocollars (equipped with a mortality sensor) to document survivorship in the wild and 11 others (seven juveniles/four adults) were euthanized with a fatal dosage of pentobarbitol (1 ml/kg) to collect data on hindlimb muscle architecture (see below). Finally, an additional 10 rabbits (nine juvenile/one adult) who did not contribute locomotor data were also euthanized to supplement the morphological dataset. Overall, our ontogenetic *S. floridanus* dataset extended over an order of magnitude in M_b_ (range: 0.106–1.434 kg).

### Performance testing

**Fig. 4:**
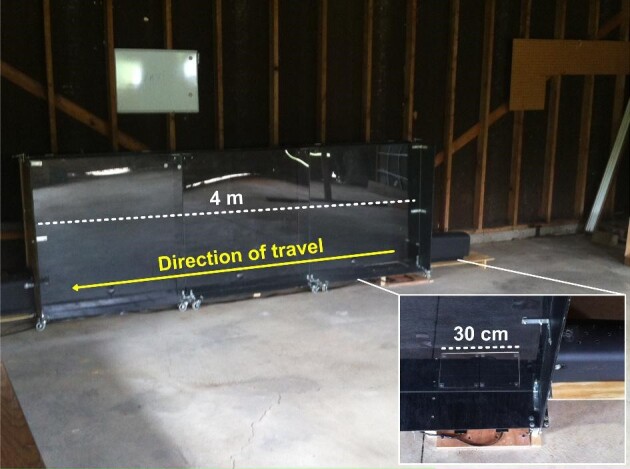
Custom constructed locomotor runway used for performance testing. Rabbits were coaxed to move across the 4-m-long runway using noxious stimuli (i.e., puffs of compressed air, loud noises, or blunt prods). Inset shows two small animal force plates used to record GRF during trials of burst acceleration. Note that force plates were mounted flush with the runway surface (mechanically isolated from the runway by a gap of 1 cm on all sides) and secured to the floor of the surrounding field station using concrete anchors. Stairway tread tape (3M Corporation, Maplewood, MN) was affixed to the top surface of the force plate to ensure rabbits had sufficient traction for acceleration.

Captured animals were transported to the Portage Park District Breakneck Creek Field Station for assessment and locomotor testing (median number of trials per animal: 15; total N: 628 trials, 195 of which were sufficient to calculate whole-body acceleration during a stride). We collected biomechanical data on burst acceleration performance in a 4-m-long enclosed runway (Fig. 4). We used a variety of noxious stimuli to elicit maximal escape responses, including puffs of compressed air, loud sounds, and blunt prods to the posterior trunk. Ground reaction forces (GRF) were sampled at 500 z using two small animal force plates (HE6 × 6-16 AMTI, Watertown, MA; sampling at 500 Hz) mounted at the start of the runway. The force platforms were secured to the concrete floor of the field station and mechanically isolated from the runway by a gap of 1 cm on all sides. Stairway tread tape (3M Corporation, Maplewood, MN) was affixed to the top surface of the force plate to ensure rabbits had sufficient traction for acceleration. Burst locomotion (sprinting from a static start) was filmed with two high-speed cameras (Fastec TS3 100-L, Fastec Imaging, San Diego, CA; sampled at 250 Hz) placed approximately 45° to the direction of travel and positioned approximately 90° relative to one another. Force platform and camera recordings were synchronized by means of a common trigger.

### Locomotor data processing

Synchronized force and kinematic data were imported in a custom-written routine in MATLAB R2019b (Mathworks, Natick, MA) routine for additional processing. The three-dimensional coordinates of the hip were fit to a quintic smoothing spline function (tolerance of 0.75 mm^2^), to mitigate digitizing error and interpolate hip position in any frames where the marker was not visible (Walker 1998). We calculated mean center of mass (COM) acceleration (a_COM_) as the quotient of mean fore-aft force and M_b_ (Roberts and Scales 2002). Escape speed (v_esc_; final velocity of the COM) was calculated as:

(1) \begin{eqnarray*} \frac{{\mathop \int \nolimits_{{t_0}}^{{t_{end}}} {F_{FA}}dt}}{{{M_b}}} + {v_0}, \end{eqnarray*}

where t_0_ and t_end_ represent the time points at the beginning and end of the stride, F_FA_ is instantaneous fore-aft force, and v_0_ is initial velocity. Initial velocity was found by using the open source motion-tracking software DLTdv6 (Hedrick 2008) to track the sagittal plane displacement of the hip marker from 10 frames (40 ms) before to 10 frames after the contact period. Initial velocity estimates were optimized using an iterative procedure that minimized the sum of the squared differences between kinetic and kinematic velocity profiles (Daley et al. 2006).

### Mechanics of acceleration

To accelerate, an animal must increase the kinetic energy of its COM over a finite interval. The work-energy theorem states that the change in kinetic energy must equal the total mechanical work performed on COM during that interval (equivalently, the product of average mechanical power and the duration over which work is performed):

(2) \begin{eqnarray*} \frac{1}{2}{M_b}\ \Delta {v^2} = {P_{COM}}\ t, \end{eqnarray*}

where Δv is the change in COM velocity, P_COM_ is average mechanical power (calculated here based on fluctuations in the mechanical energy of the COM, following Roberts and Scales (2002)), and *t* is the interval over which mechanical work is performed (i.e., the “hindlimb stroke duration”). Solving for velocity and differentiating shows that:

(3) \begin{eqnarray*} {a_{COM}} = \sqrt {\frac{{{P_{COM}}}}{{2{M_b}t}}}. \end{eqnarray*}

**Fig. 5:**
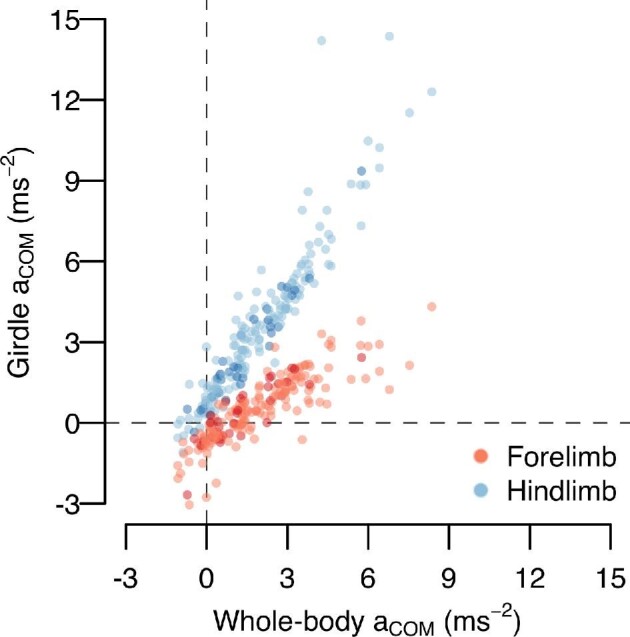
Dependence of mean whole-body acceleration over the stride (i.e., whole-body a_COM_) on mean acceleration during the period forelimb *versus* hindlimb contact (i.e., girdle a_COM_). As whole-body acceleration increased, increases in hindlimb propulsion (i.e., positive acceleration) significantly outpaced increases in forelimb propulsion (i.e., slope comparison between fore- and hindlimb acceleration versus whole-body a_COM_, *P* < 0.001). Moreover, acceleration during hindlimb contact was significantly greater than during acceleration during forelimb contact for all values of whole-body a_COM_ (*P ≤* 0.022). Finally, model comparison showed that whole-body a_COM_ was more dependent on hindlimb acceleration than forelimb acceleration (ΔAIC after removing hindlimb acceleration from the model: 325.4; ΔAIC after removing forelimb acceleration from the model: 162.3).

An animal thus has two strategies to increase a_COM_ and achieve a high v_esc_: either increase mass-specific mechanical power (P_COM_/M_b_) or decrease stroke duration (*t*). We therefore predicted that across *S. floridanus* ontogeny, acceleration would be directly proportional to the mass-specific mechanical power applied to the COM and inversely proportional to stroke duration. We specifically focused on power generation during hindlimb push-off, as preliminary analyses showed that a_COM_ was more dependent on hindlimb propulsion than forelimb propulsion (Fig. 5). In turn, we predicted that P_COM_ should be determined by the maximal power-generating capacity of the hindlimb extensor muscles (i.e., P_musc_, estimated here based upon architectural measurements of several hindlimb extensor muscles; detailed below).

### Estimated peak hindlimb extensor muscle power

We used architectural measurements of several hip, knee, and ankle extensor muscles to estimate how growth affected total available peak instantaneous hindlimb muscle power (P_musc_) during *S. floridanus* ontogeny (see Table 1 for a list of the specific muscles in our sample). Following our previously published protocol (Butcher et al. 2019), muscles were individually excised from the hindlimb and with their tendons removed, muscle belly mass (MM) was measured with an electronic balance. Muscle fascicle length (*L*^F^, in cm) and pennation angle (θ, measured as the angle between the fiber fascicles and either the long axis of the muscle or internal tendon) were measured at 5–10 random locations representative of both proximal-to-distal and superficial-to-deep regions throughout each muscle belly. Physiological cross-sectional area (PCSA, in cm^2^) was then calculated as:

(4) \begin{eqnarray*} \frac{{MM \times \cos \theta }}{{{L^F} \times \ \rho }}, \end{eqnarray*}

where *ρ* represents a standard value of density (1.06 g/cm^3^) for mammalian skeletal muscle (Méndez and Keys 1960). Maximum isometric force (*F*_max_) was estimated by multiplying PCSA by an assumed maximum isometric stress of 30 N cm^−2^ (Woledge et al. 1985). Peak instantaneous muscle power was then estimated to be one-tenth the product of *F*_max_ and *V*_max_ (Hill 1938), where *V*_max_ is maximum fiber shortening velocity (equal to 6.3 fiber lengths per second for rabbit MHC-2X muscle fibers; Pate et al. 1995). Finally, P_musc_ was calculated as twice the sum of estimated peak instantaneous power of all individual muscles (to represent simultaneous hindlimb extension during the half-bounding stride of a rabbit). We note that our calculations of P_musc_ are only estimates, indicating peak physiological muscle capacity based on architecture.

**Table 1:** Hindlimb extensor muscles sampled to estimate peak hindlimb muscle power

Hip extensors
*m. gluteus profundus*
*m. gluteus medialis*
*m. biceps femoris* (vertebral and pelvic heads)
*m. semimembranosus*
Knee extensors
*m. vastus lateralis*
*m. rectus femoris*
Ankle extensors (plantarflexors)
*m. gastrocnemius* (lateral and medial heads)
*m. soleus*

### Radio telemetry and survivorship

**Fig. 6:**
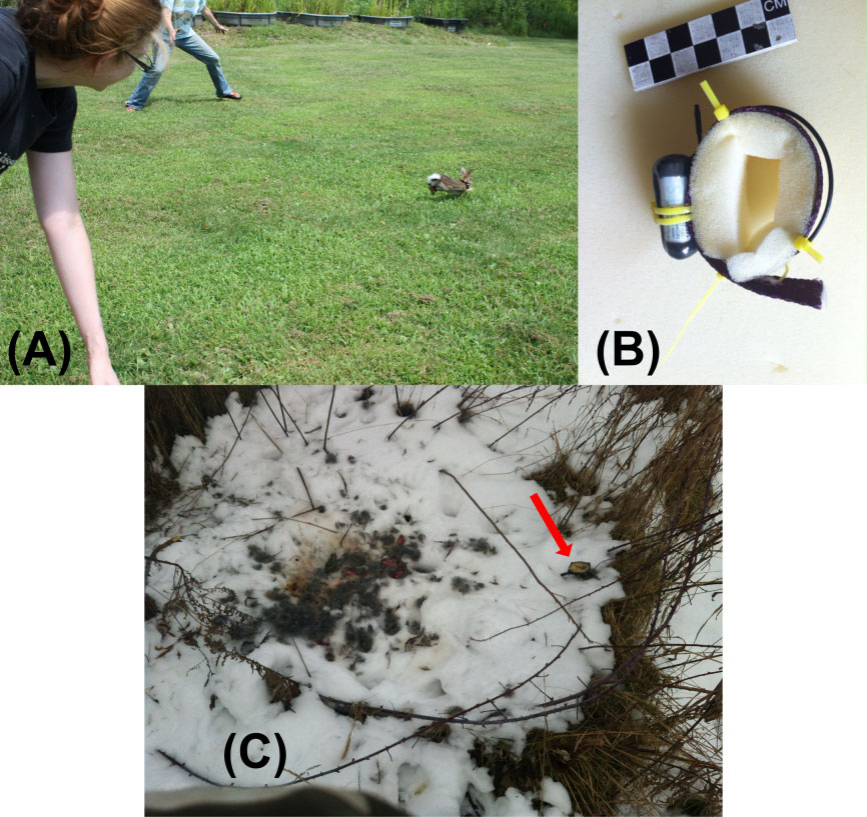
Radio-telemetry methods to document survival duration in the wild. (**A**) Field release of a 578 g juvenile rabbit (estimated age: 73 days) wearing a 20 g radio collar (i.e., 3.5% of body mass). (**B**) Custom-constructed foam-lined collar for radio-tracking juvenile rabbits; based on the design of O'Donoghue (1994). (**C**) Post-mortem retrieval of a collar fit to a 772 g juvenile rabbit. The red arrow indicates the position of the radio-collar. The remnants are consistent with carnivoran predation.

After recording body mass, but prior to locomotor testing, the subset of rabbits included in our survivorship sample were fit with radio-collars (Models 1545 and 1555, Advanced Telemetry Systems, Isanti, MN). Collar mass equaled 1.5–7.7% of the rabbit's body mass (mean: 3.0%; 95% confidence interval [CI] = 2.37$-$3.72%) (Fig. 6A). Juvenile rabbits were fit with custom constructed collars made of polypropylene nylon straps and foam, to allow for neck growth (Fig. 6B; based on O'Donoghue's (1994) design for radio-collaring juvenile snowshoe hares). Adult collars were fit with locking zip ties shielded with soft plastic sleeves.

Immediately following locomotor data collection, radio-collared rabbits were released at the site of capture. Released rabbits were checked daily using an R410 scanning receiver and a three-element folding Yagi antenna (Advanced Telemetry Systems). The radio-transmitters were equipped with mortality sensors that doubled the frequency of the transmitted pulse signal after remaining static for ≥8 h. Once we detected a mortality signal, we triangulated the rabbit's position, precisely recorded the location using a Global Positioning System receiver, photographed the scene, and retrieved the collar and any carcass material present (Fig. 6C). Because all of the rabbits in our sample eventually died, we use the duration of survival following release as our primary metric of survivorship, rather than a binary variable like survival to a certain age or calendar date, as has been the protocol of several previous studies of natural selection on whole animal performance (Irschick et al. 2008).

Across the dataset, collared rabbits exhibited significantly *greater* accelerations than uncollared rabbits (t_[277]_ = 2.18, *p =* 0.03) and, in juveniles, significantly *greater* escape speeds (t_[277]_ = 2.3, *p =* 0.024). Wearing a collar had no significant effect on escape speed in adult rabbits (t_[39]_ = −1.2, *p =* 0.24). These data suggest that the presence of a collar did not impede locomotor performance in *S. floridanus*.

### Statistical analyses

Analyses of the full locomotor performance dataset (i.e., where each individual was represented by multiple trials) were carried out using mixed-effects models (Pinheiro and Bates 2000), where individual rabbit was included as a random factor. Mixed-effects models were fit using maximum likelihood estimation to facilitate model comparison via calculation of corrected Akaike information criteria weighted by sample size (AICc). Because the residuals of such models were often characterized by heteroscedasticity, the variance of the error term was modeled as a linear function of the independent (predictor) variable. Coefficients of determination (i.e., R^2^) for these models were calculated following Johnson (2014). Analyses of reduced datasets where only a single value existed per individual (e.g., comparison of within-individual peak values between juveniles and adults), were carried out using standard single-level statistical tests (e.g., least-squares regressions and Student's *t*-tests).

We used path analysis (Li 1975) to clarify the multivariate influence of M_b_, estimated P_musc_, and mean P_COM_ on accelerative capacity during *S. floridanus* ontogeny. All variables were scaled and centered (i.e., standardized as z-scores) prior to analysis to improve parameter estimation during model fitting and make the magnitude of path coefficients directly comparable to one another. Path models were fit to the data using iterative maximum likelihood estimation (Schermelleh-Engel et al. 2003). Each path model was initially fit with all of the hypothesized paths included in the model. Non-significant paths were then removed from the model specification and the model was refit, resulting in the most parsimonious model for the data. The overall fit of the path model was evaluated via standard metrics (Schermelleh-Engel et al. 2003), i.e., a χ^2^-test comparing the empirically observed correlation matrix to the matrix implied by the model, root mean square error of approximation (RMSEA), and the comparative fit index (CFI). Non-significant χ^2^-tests (*P* > 0.05), RMSEA values not significantly different from 0.05, and CFI values ≥ 0.95 indicate acceptable fit (Schermelleh-Engel et al. 2003).

Significance for all tests was accepted at *P ≤* 0.05. For all categorical comparisons between juvenile and adults rabbits, we calculated Cohen's d as a measure of effect size (Quinn and Keough 2002), and followed Sawilosky's (2009) categorical “rules of thumb” for categorizing effects as very small, small, moderate, large, very large, and huge. All statistical analyses were conducted in the R statistical platform (version 3.4.3) (R Core Team 2018), including the add-on packages car (Fox and Weisberg 2011), dplyr (Wickham et al. 2021), emmeans (Lenth 2020), MuMIn (Bartoń 2020), lavaan (Rosseel 2012), nlme (Pinheiro et al. 2019), pwr (Champely 2018), and rptR (Stoffel et al. 2017).

## Results

### Ontogeny of locomotor performance

Acceleration performance and escape speed were significantly repeatable across the samples, as quantified by intraclass correlation coefficients (ICCs) (a_COM_ ICC: 0.359 [95% CI = 0.180-0.512]; v_esc_: ICC: 0.537 [95% CI = 0.353-0.664]). These values are comparable to ICCs observed in previous studies of animal locomotor performance (Adolph and Pickering 2008). Within individuals, trial number was unrelated to either a_COM_ or v_esc_ (*P* ≥ 0.078), demonstrating that rabbits showed no evidence of fatigue or habituation during data collection.

**Fig. 7:**
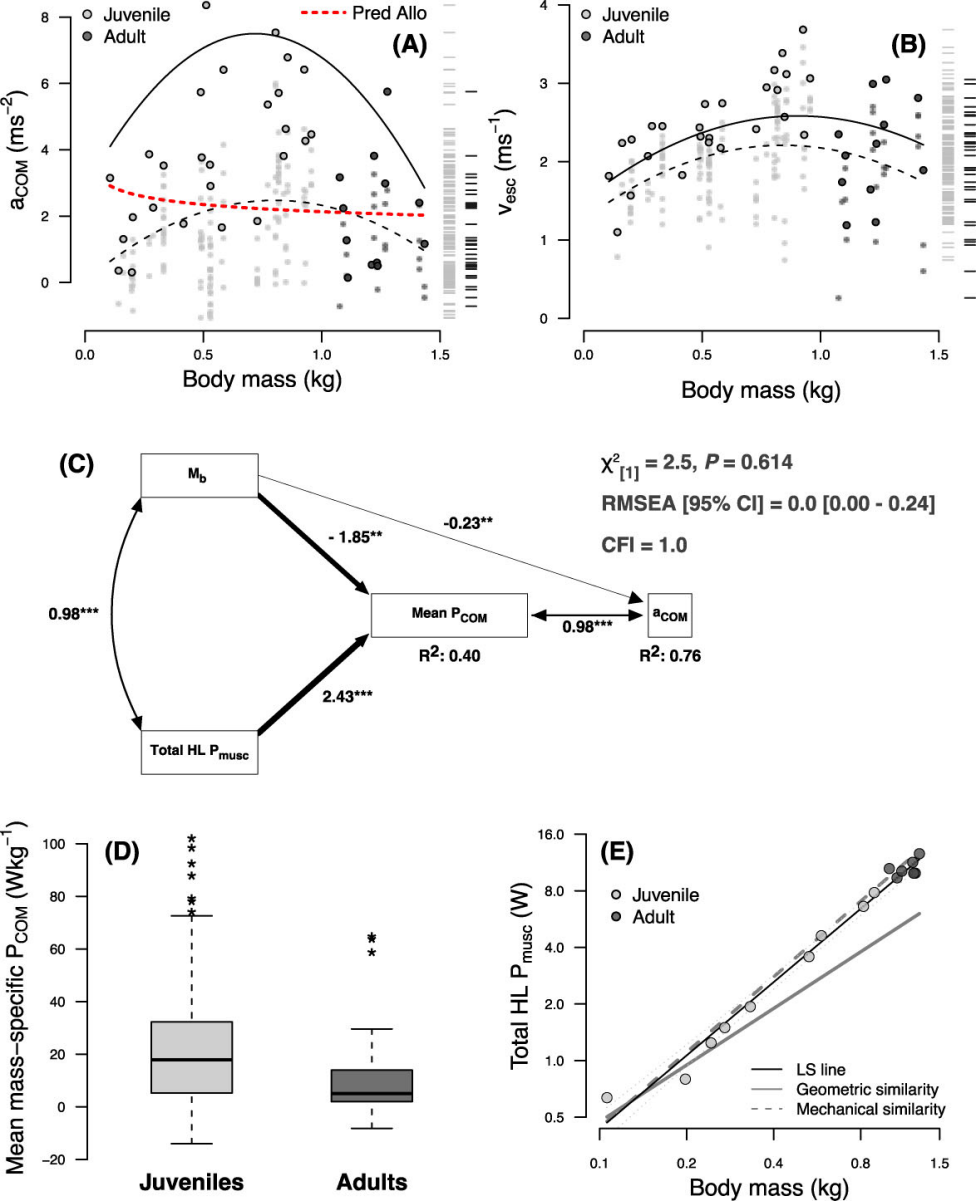
Ontogeny of locomotor performance in *S. floridanus*, showing the ontogenetic trajectory of a_COM_ (**A**) and of v_esc_ (**B**), relative to body mass. In both plots, each data point represents an individual performance trial from a single rabbit. Large symbols with bold outlines indicate the maximum value from each rabbit. The dashed trend line represents a mixed-effects quadratic regression, whereas the solid trend line represents a generalized least-squares quadratic regression through the maximum acceleration achieved by each rabbit. Rug plots to the right of each panel illustrate the distribution of performance metrics in adults and juveniles. The dashed red line in (**A**) indicates the allometric prediction of acceleration performance for a generic terrestrial running animal of similar body size (Cloyed et al. 2021). (**C**) Path analysis of the physiological and mechanical determinants of locomotor performance in *S. floridanus*. Raw variables were converted to z-scores prior to analysis, making all path coefficients standardized (i.e., equivalent to Pearson correlations coefficients for double-headed paths and β-weights for single headed paths). Path thickness is proportional to the magnitude of the association between the indicated variables. *P*-values correspond to the significance of individual path coefficients (*** *P* < 0.001, ** *P* < 0.01). Multivariate coefficients of determination (i.e., R^2^) values are provided for all endogenous variables. Hindlimb stroke duration (t) was not significantly associated with variation in a_COM_ once other variables were considered and was therefore not included in the final model. Fit metrics all indicated an acceptable model fit, i.e., a non-significant χ^2^-test, a RMSEA value ≤0.05, and a CFI values ≥0.95. (**D**) Box-and-whisker plots of mean mass-specific power production in juvenile and adult rabbits. Dark lines represent the median of each distribution, boxes extend across the interquartile range, and whiskers extend to ±150% of the interquartile range. Asterisks indicate outliers beyond this range. (**E**) Ontogenetic allometry of estimated peak instantaneous hindlimb extensor muscle power in *S. floridanus*. The thin black solid line indicates the least-squares regression fit (in log-log space) of P_musc_ versus M_b_, with the adjacent dotted lines indicating the 95% CI about the fitted line. The solid gray line indicates the scaling prediction under geometric similarity (i.e., P_musc_ ∝ M_b_^1.00^) and the dashed gray line indicates the scaling prediction under mechanical similarity (i.e., P_musc_ ∝ M_b_^1.33^).

Juveniles were capable of producing greater accelerations than adults (Fig. 7A), though mean differences did not reach statistical significance (F_[1,36]_ = 2.22, *P =* 0.145, Cohen's d = 0.25 [small effect]). Nevertheless, peak accelerative performance was clearly dominated by juveniles. Maximal accelerations recorded within individuals were significantly greater in juveniles than adults (t_[27.4]_ = 2.3, *P =* 0.007; Cohen's d = 1.27 [very large effect]). The ontogenetic trajectories of both mean and peak a_COM_ were best modeled as quadratic functions of body mass (Table 2; log-likelihood tests comparing linear *versus* quadratic models: *P ≤* 0.014). Overall, peak acceleration capacity was greatest during the late juvenile period at a model-predicted body mass of 0.74 kg and an estimated age of 94 days (95% confidence bounds: 90.3, 97.0 days).

**Table 2:** Regression models of locomotor performance ontogeny in *S. floridanus*

Model	Statistic^1^	*P*	AICc^2^	R^2^
1. Mean a_COM_ ∼ body mass				
Linear model	χ^2^_[1]_ = 1.15	0.283	751.4	0.011
Quadratic model	χ^2^_[2]_ = 9.25	0.010	747.5	0.272
2. Peak a_COM_ ∼ body mass				
Linear model	F_[1,36] =_ 0.04	0.851	173.1	<0.001
Quadratic model	F_[1,35] =_ 14.5	<0.001	162.4	0.294
3. Mean v_esc_ ∼ body mass				
Linear model	χ^2^_[1]_ = 0.87	0.351	281.3	0.011
Quadratic model	χ^2^_[2]_ = 6.84	0.033	277.4	0.100
4. Peak v_esc_ ∼ body mass				
Linear model	F_[1,36] =_ 1.05	0.312	73.4	0.028
Quadratic model	F_[1,35] =_ 9.29	0.004	67.5	0.232

1 Tests of the overall statistical significance of the model, versus an intercept-only model. We used likelihood-ratio tests to evaluate mixed-effects fits (i.e., models 1 and 3) and F-tests to evaluate the least-squares models (i.e., models 2 and 4).

2 Akaike information criterion, adjusted for sample size.

Mean a_COM_ was significantly positively associated with v_esc_ at the end of the stride, both on a trial-to-trial basis (*P* < 0.001; R^2^ = 0.64), and when restricted to maximal performance (*P* < 0.001; R^2^ = 0.60). The enhanced acceleration capacity of juvenile rabbits allowed them to reach adult-like escape speeds (Fig. 7B), despite being absolutely smaller in body size. Neither mean nor peak v_esc_ significantly varied between age categories (all *P ≥* 0.131). Nevertheless, the ontogenetic trajectory of both mean and peak v_esc_ were best described by quadratic models (Table 2; log-likelihood tests comparing linear *versus* quadratic models: *P ≤* 0.015), indicating that escape speeds were maximal during the juvenile period. Based on the quadratic regression model, peak v_esc_ is predicted to be greatest during the late juvenile period, at a body mass of 0.82 kg and an estimated age of 104 days (95% confidence bounds: 101.0, 107.8 days).

### Physiological and mechanical determinants of locomotor performance

Corroborating predictions, path analysis showed that acceleration performance was directly associated with mean P_COM_ and negatively associated with M_b_. Together, mean P_COM_ and M_b_ explained 76% of the variation in acceleration performance across the ontogenetic dataset (Fig. 7C). Mean P_COM_ was positively associated with total estimated hindlimb P_musc_ (controlling for M_b_) and negatively associated with body mass (controlling for total hindlimb P_musc_), with these two variables explaining 40% of the variation in mean P_COM_ over the ontogenetic dataset. Because hindlimb stroke duration was not significantly associated with a_COM_ once variables were considered, models incorporating hindlimb stroke duration resulted in an overall poorer fit. For this reason, hindlimb stroke duration was not included in the final path model presented here.

As suggested by this path model, mass-specific power outputs were significantly greater in juveniles than in adults (Fig. 7D; t_[40]_ = 4.1, *P <* 0.001; Cohen's d = 0.65 [moderate effect]). Nevertheless, ontogenetic scaling analysis showed that hindimb P_musc_ scaled to body mass at a rate needed to maintain “mechanical similarity” (i.e., maintenance of functional capacities across growth-related increases in size; Carrier 1983) (Fig. 7E; P_musc_ = 2.2M_b_^1.32 [1.240, 1.404]^, R^2^: 0.98; mechanical similarity prediction = M_b_^1.33^; also see Butcher et al. (2019)), indicating that the superior mass-specific power capacities of juvenile rabbits were more likely due to their smaller overall size than more powerful muscles *per se*.

### Locomotor performance and survival duration

**Fig. 8:**
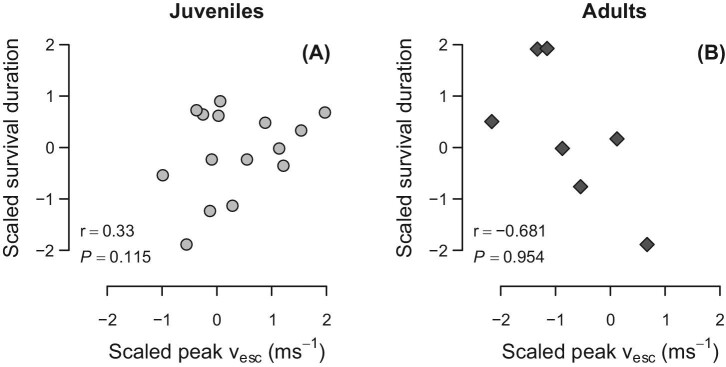
Association between peak v_esc_ and survival duration in (**A**) juvenile and (**B**) adult *S. floridanus*. Raw values were Box-Cox transformed and converted to z-scores to improve normality (Box and Cox 1964; Sokal and Rohlf 1995). Reported statistics represent one-tailed Pearson product-moment correlations testing for a positive relationship between survival duration and peak v_esc_.

Given that selective pressure to avoid predation should be greatest on juveniles (Fig. 1), we predicted that maximum escape speed should be directly associated with the duration of survival in juvenile rabbits, but not necessarily in adults. One-tailed Pearson correlation tests showed that survival duration after release was positively associated with peak v_esc_ in juvenile *S. floridanus*, though the association did not reach statistical significance (Fig. 8A; *P =* 0.115). In contrast, survival duration was not positively associated with peak v_esc_ in adults (Fig. 8B).

## Discussion

### Locomotor performance in S. floridanus

Juvenile *S. floridanus* accelerate more rapidly than adults. The highest acceleration in the dataset (8.36 ms^−2^), recorded from an 856 g juvenile rabbit with an estimated age of 110 days, rivals peak values previously reported from more iconic running mammals, including polo ponies, greyhound dogs, and cheetahs (i.e., values of 6–12 ms^−2^) (Williams et al. 2009; Wilson et al. 2014), as well as sauropsid taxa of roughly similar size to cottontail rabbits; i.e., green iguanas (body mass: 1.37 kg; maximal acceleration: 4.85 ms^−2^) (Scales and Butler 2007) and wild turkeys (body mass: 3.08 kg; maximal acceleration: 5.3 ms^−2^) (Roberts and Scales 2002), and is greater than the predicted acceleration performance for a generic running animal of similar body size (Fig. 7A; Cloyed et al. 2021).

Biomechanical modeling has demonstrated that mammalian acceleration should be broadly limited by two factors: (1) the need to avoid the potentially destabilizing nose-up pitching torques that result from large, unbalanced propulsive GRF and (2) the amount of mechanical power locomotor muscles can produce (Williams et al. 2009). The characteristic half-bounding gait of cottontail rabbits and other leporids may help them circumvent both limitations. Any potential nose-up pitching torques that might destabilize other mammals would instead be used to launch the rabbit into the next flight phase. Additionally, the synchronous extension of both hindlimbs during bounding effectively maximizes power generation relative to gaits with asynchronous limb contacts (such as trotting and galloping).

**Fig. 9:**
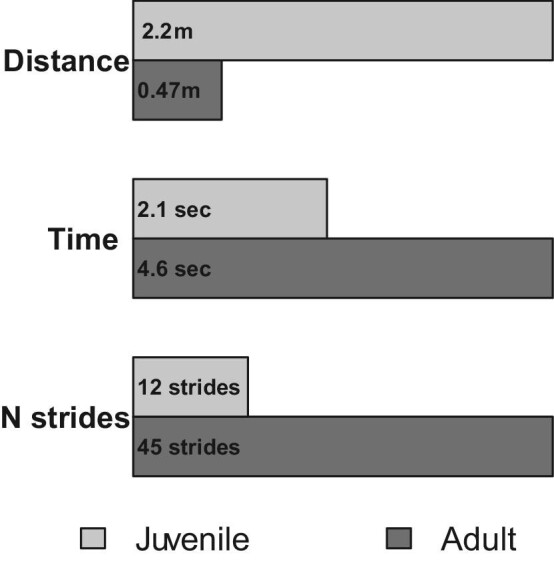
Functional implications of ontogenetic variation in locomotor performance. Bar graphs represent the predicted performance of a juvenile rabbit (0.8 kg in body mass) and an adult rabbit (1.2 kg in body mass) under three hypothetical scenarios of constant acceleration from a static starting position: (1) distance travelled during the first second of sprinting, (2) time required to reach a refuge 10 m away, and (3) number of strides required to reach 11.1 ms^−1^, the maximal speed recorded for *S. floridanus* (Garland 1983).

From a life history perspective, both acceleration capacity and escape speed followed an inverted parabolic trajectory, peaking during the late juvenile stage of life, a period when rabbits are dispersing from the natal nest and first experiencing a pronounced episode of demographic attrition (Fig. 1). Given that most *S. floridanus* fatalities in the wild are due to predation (Keith and Bloomer 1993; Fritsky 2006; Boland and Litvaitis 2008), we would expect selective pressures on locomotor performance during this time to be particularly acute. To explore the possible ecological implications of this inverted parabolic performance trajectory in cottontail rabbits, we used the quadratic regression of maximal accelerations (Fig. 7A) to model juvenile and adult locomotor performance during three hypothetical scenarios (all assuming constant acceleration): (1) distance travelled in the first second of sprinting from a standing start, (2) time required to reach a refuge 10 m away, and (3) number of strides required to reach 11.1 ms^−1^, the top recorded speed for *S. floridanus* (Garland 1983). In each scenario, the juvenile out-performed the adult—travelling 163% further during the first second of sprinting, requiring only 79% of the adult's travel time needed to reach a refuge 10 m away, and reaching maximal running speed in 11 fewer strides (Fig. 9).

### Ontogenetic determinants of performance

Our analyses suggested several mechanical relationships that may help explain the superior locomotor performance of juvenile *S. floridanus*. The lower body mass of juvenile rabbits improves accelerative performance both directly and indirectly via the negative influence of body mass on mean P_COM_ (when controlling for total hindlimb P_musc_) (Fig. 7C). As a result, juvenile rabbits are able to outpace adults by producing more mechanical power per unit body mass. Previous studies of acceleration performance in turkeys, wallabies, and greyhound dogs have also shown a tight association between acceleration and mass-specific power production (Roberts and Scales 2002; McGowan 2005; Williams et al. 2009). By analogy, a similar metric—the power-to-weight ratio—is typically used to evaluate the performance capacities of automobiles, where performance can be improved by either increasing engine power or decreasing weight. Juvenile rabbits of course weigh less than adults, but what can be said about the power of their “engines” (i.e., hindlimb extensor musculature) relative to adults? Since ontogenetic scaling analyses suggested that rabbits of all sizes should be capable of functionally similar levels of muscle power (i.e., P_musc_ scaled to M_b_ with mechanical similarity, Fig. 7E), the superior performance of juveniles is most likely due to their absolutely smaller body size. Seen in this light, the ontogenetic allometry of the *S. floridanus* musculoskeletal system may be more indicative of the need for adults to keep pace with juveniles, rather than the converse (Butcher et al. 2019).

Alternatively, the lower locomotor performance of adult rabbits could be an example of “behavioral compensation”, whereby adults have acquired additional strategies of predator avoidance (i.e., greater crypsis, adjustments to activity patterns, etc.), and are therefore able avoid sprinting at their maximum capacity (Dial et al. 2008), perhaps allowing them to conserve metabolic energy. Irschick (2000) documented this phenomenon in *Anolis* lizards, showing that juveniles typically sprint nearer to their peak capacities than do adults. Given the demographic curve of *S. floridanus* (Fig. 1), any cottontail rabbit that has reached adulthood has undoubtedly endured an intense period of selection and gained valuable experience about performance thresholds and alternative means of predator evasion in the process.

### Natural selection on locomotor performance

The predicted age range of peak locomotor performance (i.e., 3–4 months of age) closely corresponds to the ages at which developing rabbits first experience pronounced demographic attrition (Fig. 1). As such, we predicted that the juveniles that achieved the greatest escape speeds would survive for longer durations in their natural habitats by being better able to avoid predation. However, we found that although survival duration following capture was positively associated with v_esc_, the positive correlation was not statistically significant. As we discuss below, this may be partly due to an insufficient sample size in our radio-collared subsample of rabbits.

### Limitations

It is possible that the 4-m length of our experimental trackway constrained acceleration performance in the oldest, largest rabbits. However, there are a few reasons to think that this was not the case. First, as noted above, our data collection protocol during performance testing was designed to elicit maximal escape responses from captured rabbits. Second, the peak acceleration values sampled for the largest rabbits are close to predicted values from a recent allometric study of acceleration performance in running vertebrates (Fig. 7A; Cloyed et al. 2021). Third, a shorter testing track length may represent ecologically realistic experimental design for testing locomotor performance in *S. floridanus*. As stated above, predator evasion in cottontail rabbits is chiefly accomplished via quick acceleration to nearby dense vegetation (Trent and Rongstad 1974; Cowan and Bell 1986; Temple 1987; Vidus-Rosin et al. 2010).

To some degree, our inability to detect a significant association between performance and survivorship may be due to limited statistical power. The observed power in testing for correlations between v_esc_ and survival duration in juvenile rabbits was 0.33. Assuming a similar effect size, obtaining a power level of 0.8 would have required sampling an additional 41 juvenile rabbits. Given the multitude of ecological factors influencing survivorship and other metrics of Darwinian fitness, empirically demonstrating natural selection on phenotypic and performance-related traits in wild animals has been shown to be notoriously difficult, often requiring sample sizes of several hundred animals (Jayne and Bennett 1990; Kingsolver et al. 2001; Miles 2004; Husak 2006; Irschick et al. 2008). Finally, it is possible that other unmeasured aspects of locomotor performance, such as maneuverability/agility (e.g., peak capacity for centripetal acceleration) may be more closely tied to predator evasion and survival duration in cottontail rabbits than linear acceleration and sprint speed per se (Clemente and Wilson 2015; Moore and Biewener 2015; Wilson et al. 2018), and multivariate assessments of performance may ultimately prove to be more informative.

## Conclusions

We have shown that locomotor performance peaks during the late juvenile period of life in *S. floridanus.* Our results have implications for understanding how natural selection should broadly influence performance phenotypes. From a Darwinian perspective, juvenility is inherently maladaptive—all else being equal, natural selection should favor rapid growth and development to reproductive maturity (Williams 1966; Promislow and Harvey 1990). However, rapid growth can itself entail additional costs to the developing organism. Growth is metabolically expensive and any hastening in the pace of growth necessarily requires the animal to either obtain additional energetic resources or shunt energy away from other critical metabolic functions (Case 1978; Promislow and Harvey 1990). Additionally, rapid differentiation of biological tissues potentially degrades their quality (e.g., woven bone can be laid down more quickly than lamellar bone, but is also mechanically weaker; Carrier 1996; Currey 2002). Alongside rapid growth, natural selection should thus favor heightened juvenile performance as a means of negotiating ontogenetic constraints on survivorship, particularly when predation pressure is high. Indeed, the few studies that have examined associations between whole-organism performance and survivorship have predominantly found that directional selection on locomotor performance is more often manifest in juvenile animals than in adults (Jayne and Bennett 1990; Le Galliard et al. 2004; Miles 2004; Husak 2006; Irschick et al. 2008). By corroborating and extending previous studies of performance ontogeny (Carrier 1996; Herrel and Gibb 2006; Irschick et al. 2008), we have demonstrated that juvenile cottontail rabbits are marked by increased mechanical power capacities, facilitating superior locomotor performance. Age-specific variation in mortality risk should be considered as an important factor when evaluating how selection operates on whole-organism performance and the underlying morphological traits.

## Data Accessibility

Datasets used for all analyses in this study are publicly available on figshare at https://doi.org/10.6084/m9.figshare.6815996.
